# Evaluation of midkine and anterior gradient 2 in a multimarker panel for the detection of ovarian cancer

**DOI:** 10.1186/1756-9966-29-62

**Published:** 2010-06-03

**Authors:** Gregory E Rice, Tracey A Edgell, Dominic J Autelitano

**Affiliations:** 1 HealthLinx Ltd, Richmond, Victoria, Australia

## Abstract

The aims of this study were: to characterise and compare plasma concentrations of midkine (MDK) in normal healthy women with concentrations observed in women with ovarian cancer; and to establish and compare the performance of MDK with that of anterior gradient 2 protein (AGR2) and CA125 in the development of multi-analyte classification algorithms for ovarian cancer. Median plasma concentrations of immunoreactive MDK, AGR2 and CA125 were significantly greater in the case cohort (909 pg/ml, 765 pg/ml and 502 U/ml, respectively n = 46) than in the control cohort (383 pg/ml, 188 pg/ml and 13 U/ml, respectively n = 61) (p < 0.001). The area under the receiver operator characteristic curve (AUC) for MDK and AGR2 was not significantly different (0.734 ± 0.046 and 0.784 ± 0.049, respectively, mean ± SE) but were both significantly less than the AUC for CA125 (0.934 ± 0.030, p < 0.003). When subjected to stochastic gradient boosted logistic regression modelling, the AUC of the multi-analyte panel (MDK, AGR2 and CA125, 0.988 ± 0.010) was significantly greater than that of CA125 alone (0.934 ± 0.030, p = 0.035). The sensitivity and specificity of the multi-analyte algorithm were 95.2 and 97.7%, respectively. Within the study cohort, CA125 displayed a sensitivity and specificity of 87.0 and 94.6%, respectively. The data obtained in this study confirm that both MDK and AGR2 individually display utility as biomarkers for ovarian cancer and that in a multi-analyte panel significantly improve the diagnostic utility of CA125 in symptomatic women.

## Introduction

Each year, more than 200,000 women are diagnosed with ovarian cancer. Ovarian cancer is the 8^th ^most common cancer in women and the 2^nd ^most common type of gynecological cancer in the world. In the USA, the prevalence of ovarian cancer in postmenopausal women is 1 in 2,500 and the lifetime risk of a woman developing ovarian cancer is 1 in 72. The age-adjusted incidence and death rates for ovarian cancer are 13.3 and 8.8 per 100,000, respectively. The average five-year survival rate for ovarian cancer patients is ~46%. This high overall mortality is a consequence of a failure to detect this disease at an early stage. As there are no clinically overt early symptoms, most women (~75%) are first diagnosed with disseminated disease (Stage III/IV) when prognosis is poor. Despite recent progress in chemotherapeutic treatments, the diagnosis of late stage disease is associated with a five-year survival rate of ~30%. In contrast, when ovarian cancer is identified at an early stage, five year survival increases to ~90%. Thus, the development of more accurate and earlier detection tests for this disease are undoubtedly the number one priority for achieving long-term reduction of mortality from ovarian cancer [1].

Currently, plasma or serum CA125 concentration is the best characterised and most widely used ovarian cancer biomarker and is elevated in more than 80% of patients with epithelial ovarian cancer [2]. CA125 concentrations, however, are increased in only ~ 50% of patients with Stage I disease [3]. Thus, more accurate and earlier detection tests are requisite to reducing the mortality associated with this disease.

Previously, we and others have reported the utility of combining biomarkers to develop classification algorithms for identifying women with ovarian cancer [4-10]. Such studies establish proof-of-concept and the potential to improve diagnostic efficiency by combining multiple ovarian cancer biomarkers. The sensitivity and specificity of such panels, however, must be further improved and additional informative biomarkers that contribute to multivariate modelling need to be identified.

The purpose of this study was to characterise changes in the plasma concentrations of MDK in association with ovarian cancer and compare its diagnostic performance (as assessed by the AUC) with that of AGR2 (a recently reported circulating biomarker of ovarian cancer [11]) and CA125 in symptomatic women. Available data are consistent with a putative role for both AGR2 and MDK in oncogenesis and tumor progression, including ovarian cancer.

## Materials and methods

### Control and ovarian cancer plasma samples

Plasma samples were collected from healthy women (median age 52, range 32-69 years, n = 61) and women at the time of diagnosis of ovarian cancer and before treatment (median age 61, range 24-69 years, n = 46). The project was approved by the Mercy Hospital for Women Human Research and Ethics Committee (R09/06). All case samples and part of the control sample set used in this study were provided by the Biobank at Peter MacCallum Cancer Research Institute (Melbourne, Australia) and all subjects participated in the study after signing an informed written consent. Blood (10 ml) was collected via vena puncture into EDTA vacutainer tubes and samples were centrifuged at 1000 g for 10 min within 20-30 min of collection. Plasma was stored as 250-1000 μl aliquots at -80°C until assayed. Ovarian tumor classification was based on the FIGO staging system, however, no stage IV tumors were identified for inclusion in this study.

### Study Design

The study was a retrospective, case-control design (*i.e. *a phase 2 biomarker trial [12] involving 107 plasma samples (see Table 1) obtained from 61 controls and 46 cases (*i.e. *women previously diagnosed with ovarian cancer). Inclusion and exclusion criteria are presented in Table 2. The primary outcomes of the study were: quantification of plasma concentrations of ir MDK and evaluation of its diagnostic performance (as defined by AUC); and comparison with AGR2 and CA125 concentrations measured in the same cohort. In addition, the contribution for these biomarkers to multi-analyte classification models was determined.

**Table 1:** Distribution of Ovarian Tumor Types and Stages of ovarian cancer patients used for plasma AGR2 and CA125 measurements.

	All Tumors	Stage I	Stage II	Stage III	Unstaged
**Serous**	29	3	17	9	

**Mucinous**	5	1	3	1	

**Endometrioid**	4	2	2		

**Clear Cell**	2		1		1

**Mixed Mullerian**	3	1	2		

**Untyped**	3	2	1		

**Total**	46	9	26	10	1

**Table 2:** Inclusion and exclusion criteria for inclusion of patient samples in the study.

Inclusion Criteria	Exclusion Criteria
Age 18-80	Chemotherapy, biologic therapy or any other investigational drug for any reason within 28 days prior to sampling.

Newly diagnosed, histologically or pathologically confirmed diagnosis of epithelial carcinoma of the ovary.	Except for cancer-related abnormalities, patients should not have unstable or pre-existing major medical conditions.

No NSAID or prednisone use within 14 days of sampling.	Major surgical procedure, open biopsy, or significant traumatic injury within 28 days prior to sampling

No previous chemotherapy or radiation therapy.	Minor surgical procedures, fine needle aspirations or core biopsies within 7 days prior to sampling

No concurrent disease(s)	Serious, non-healing wound, ulcer, or bone

Signed informed client consent	

### Biomarker Quantitation

Plasma concentrations of MDK were quantified by sandwich ELISA assay (Peprotech, Rocky Hill, NJ, USA) that utilises rabbit antibodies raised to human midkine for both capture and detection stages of the assay. The assay was performed in Costar half-well immunoassay plates (Corning) coated with 50 μl of capture antibody at a concentration of 1 ug/ml in 50 mM carbonate buffer and incubated at 4°C for 18 h. Following four washes in PBS/Tween20 (Sigma), the plate was blocked with 150 μl/well of 0.1% BSA (Sigma) in PBS, for one hour at room temperature. Plasma samples diluted 1:2 in PBS containing 0.05% Tween20 and 0.1% BSA were applied to the plate following blocking, alongside a standard curve, from 2000 pg/ml down in doubling dilutions, constructed from a stock recombinant midkine. Samples and standards were incubated (50 μl/well) at room temperature for 2 h following which the plate was washed a further three times with wash buffer. Detection of bound midkine was made using 50 μl/well of biotinylated detection antibody at a concentration of 1.0 ug/ml for 2 h at room temperature. Following a further four washes the plate was incubated with a 1:2000 dilution of avidin-HRP conjugate for 30 min. Finally the plate was washed four times and 100 μl of OPD substrate added to the wells and incubated for 30 min in the dark. Prior to reading on a Multiskan Ascent the reaction is topped by addition of 25 μl of 3 M sulphuric acid.

AGR2 concentrations were quantified using an in-house sandwich ELISA employing a mouse monoclonal antibody (7A10) to a peptide epitope (KPGAKKDTKDSRPKL) of AGR2 that displays no measurable cross reactivity with AGR3, as previously reported [11].

CA-125 was quantified using Roche CA-125 Elecsys II assay (Roche, Mannheim, Germany, LD = 0.6 U/ml; intra- and inter-assay coefficients of variation CV = 3.3% and 4.3%) as previously reported [8].

### Statistical Analyses

Two sample group comparisons of median values were assessed by Mann Whitney tests (STAT 9.2, Stata Corporation, College Station, TX, USA). Correlation between two sample groups was assessed by Spearman's rank correlations using the Bonferoni correction). Multiple group comparisons were assessed by Kruskal-Wallis tests [13]. Dunn's tests [14] were used for post-hoc two sample comparisons. A p value of < 0.05 was ascribed as statistically significant.

### Multivariate Modelling

Binomial classification algorithms were generated, based upon biomarker data obtained in this study, using a boosted logistic regression algorithm with Weka Data Mining Software (Ver 3-6-1, [15,16]). The predicted posterior probability values reported (*i.e. *the likelihood that a sample came from a woman with ovarian cancer, that is ρP) were used to generate receiver operator characteristic curves. Sensitivity and specificity were calculated based on the numbers of correctly and incorrectly classified samples. For classification of samples based on conventional plasma CA-125 concentrations, a threshold value of ≥ 35 U/ml was used as indicative of ovarian cancer.

### ROC Curve Comparisons

For individual biomarkers, plasma concentration data were used to generate ROC curves (MedCalc, MedCalc Software bvba, Mariakerke Belgium). AUCs were calculated using the Wilcoxon statistic [17]. The diagnostic performance of the biomarkers was assessed by comparison of the area under ROC curves using the method of Hanley and McNeil [18] for ROCs derived from the same cases. A threshold value of 0.500 was used for classification of samples based on ρP. Values of > 0.500 being classified as ovarian disease and samples with a calculated value < 0.500 being classified as normal.

## Results

### Cohort Characteristics

The median age (range) of the control and case cohort were 52 years (32 - 69, n = 61) and 61 years (24 - 81, n = 46), respectively. The distribution of type and stage of ovarian cancer within the case cohort is presented in Table 1. Of the cases included in this study, 76% (*i.e. *35 cases) were early stage disease (*i.e. *Stages I and II). The median CA125 plasma concentrations were 13 U/ml (range 3 - 84) for controls and 502 U/ml (5 - 10,209) for cases. In 3 controls, CA125 concentration was ≥ 35 U/ml. In 6 cases, CA125 concentration was < 35 U/ml. At a threshold of 35 U/ml, the sensitivity and specificity of CA125 were 87.0 and 95.1%, respectively.

### Variation with Disease State, Stage and Tumor Type

**Figure 1:**
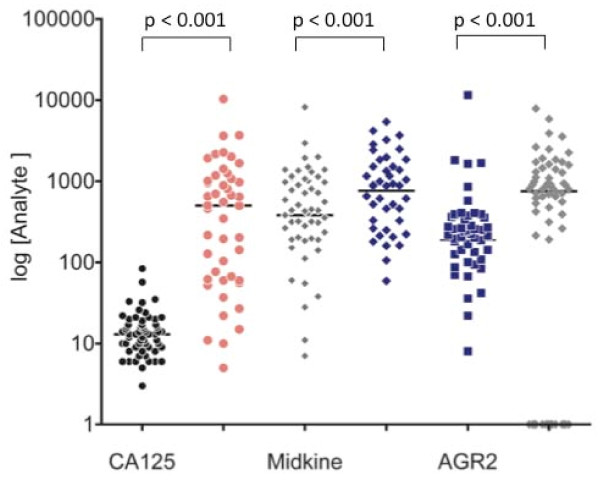
**Plasma biomarker concentrations**. The median plasma concentration within each group (normal women (controls) n = 61 and women with ovarian cancer (cases) n = 46) is represented by the horizontal line. Biomarker concentrations were significantly greater in case cohorts (solid symbols) when compared to their respective control cohort (open symbols) (p < 0.001, Mann Whitney tests). Data are presented as log (plasma concentration). CA125 as U/ml; and MDK and AGR2 as pg/ml.

The variation in plasma analyte concentrations for control and case cohorts is presented in Figure 1. Median plasma concentrations of immunoreactive MDK, AGR2 and CA125 were significantly greater in the case cohort (909 pg/ml, 765 pg/ml and 502 U/ml, respectively n = 46) than in the control (383 pg/ml, 188 pg/ml and 13 U/ml, respectively n = 61) cohort (p < 0.001, as assessed by Mann Whitney tests). Within control or case cohorts, plasma concentrations of AGR2 displayed no significant correlations with either CA125 or midkine concentrations (as assessed by Spearman's correlation, p > 0.05). Within the case cohort, MDK plasma concentrations significantly correlated with CA125 concentrations (ρ = 0.383, p < 0.01). Data were further analysed with respect to tumor type and Stage (Table 3). No statistically significant effects of either tumor type or stage on biomarker plasma concentrations were identified (Kruskal-Wallis one-way analysis of variance, p > 0.05).

**Table 3:** Case cohort variation in plasma analyte concentration by stage of disease and tumor type, as assessed by Kruskal-Wallis One Way Analysis of Variance (Stage and Tumor Type).

Analyte	**Stage n = 45**^**# **^**(p)**	**Tumor Type n = 43**^**† **^**(p)**
MDK	0.722	0.839

AGR2	0.776	0.334

CA125	0.524	0.214

# 1 sample was unstaged

† 3 samples were not typed

### Receiver Operator Characteristic Curve Analysis and Multi-analyte Modelling

ROC curves were generated for each individual analyte. The area under the curve (AUC) for MDK, AGR2 and CA125 was: 0.753 ± 0.049; 0.768 ± 0.048; 0.934 ± 0.027, respectively (AUC ± SEM). There was no significant difference between the AUC for midkine and AGR2. The AUC for CA125 was significantly greater than that for both midkine and AGR2 (p < 0.001, Table 4).

**Table 4:** Comparison of AUC for MDK, AGR2, CA125 and multi-analyte panel Data represent AUC ± standard errors (SEM).

Analyte	AUC ± SEM	p
CA125	0.934 ± 0.027	

MDK	0.753 ± 0.049	< 0.001

AGR2	0.768 ± 0.048	= 0.001

Multi-analyte Algorithm	0.988 ± 0.011	= 0.038

The difference in the AUC of MDK, AGR2 and the multi-analyte algorithm are compared to that of CA125. The AUC of MDK was not statistically significantly different from the AUC of AGR2 (p > 0.05).

**Figure 2:**
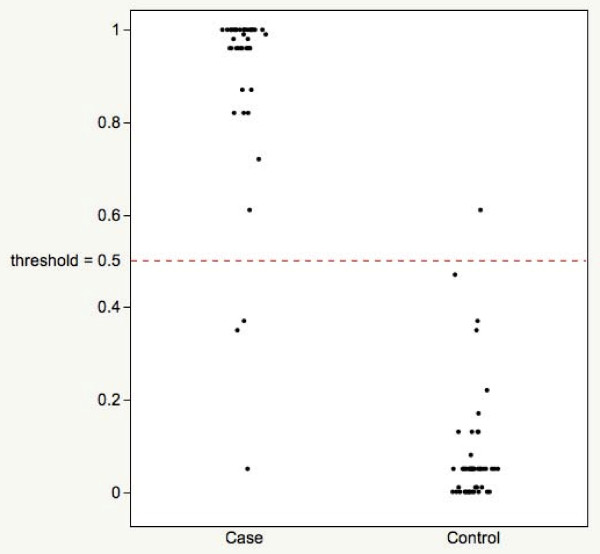
**Predicted posterior probability values (ρP)**. Values were generated by multivariate modelling for each patient set of biomarkers for Case and Control cohorts.

**Figure 3:**
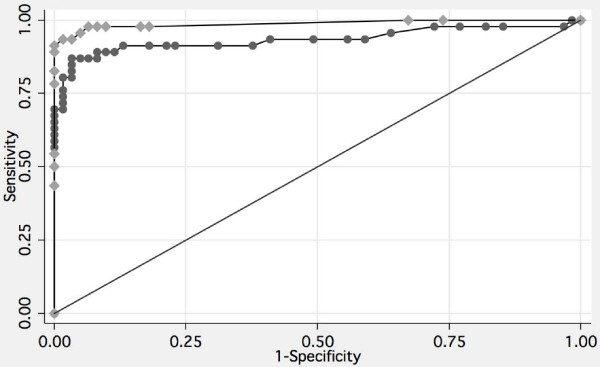
**ROC curve comparison**. ROC curves are displayed for the multi-analyte algorithm (midkine, AGR2 and CA125) and CA125 alone. The AUC (± SEM) for the multi-analyte panel (black diamond) (0.988 ± 0.010) was significantly greater than that of CA125 alone (black circle) (0.934 ± 0.030, p = 0.035).

A binomial classification algorithm was developed by subjecting the observed plasma concentrations for MDK, AGR2 and CA125 to stochastic gradient boosted logistic regression analysis [19]. A ρP value was calculated for each patient set of biomarkers and used to generate a ROC curve (Figure 2). The AUC for the multi-analyte panel (0.988 ± 0.011) was significantly greater than that for MDK (p < 0.001), AGR2 (p = 0.001) and CA125 (p = 0.038) (Figure 3). The sensitivity and specificity of the multi-analyte algorithm were 95.2 and 97.7%, respectively. Within the study cohort, CA125 displayed a sensitivity and specificity of 87.0 and 94.6%, respectively.

## Discussion

The aims of this study were: to characterise and compare plasma concentrations of midkine (MDK) in normal healthy women with concentrations observed in women with ovarian cancer; and to establish and compare the performance of MDK with that of anterior gradient 2 protein (AGR2) and CA125 in the development of multi-analyte classification algorithms for ovarian cancer. A retrospective, case-control study was conducted to compare the diagnostic performance (as measured by AUC) of plasma ir MDK and ir AGR2 individually or in combination with CA125 with the performance of CA125 alone. Biomarker plasma concentrations were quantified in normal healthy women and women with confirmed ovarian cancer. The data obtained confirm the utility of both MDK and AGR2 as plasma biomarkers for ovarian cancer and, when combined in a multi-analyte panel, significantly improve the diagnostic efficiency of CA125.

The median plasma concentrations of both ir MDK and ir AGR2 were significantly greater in women with ovarian cancer (909 pg/ml and 765 pg/ml, respectively n = 46) than in normal healthy women (383 pg/ml and188 pg/ml, respectively n = 61) (p < 0.001). There is a paucity of data characterising the plasma concentrations of MDK in ovarian cancer patients. Salama *et al. *(2006) [20] reported a similar change in serum MDK concentrations in 15 women with ovarian carcinoma (*i.e. *> 500 pg/ml) and 49 controls (*i.e. *< 500 pg/ml) to those concentrations reported in this study. Within the present study cohort, plasma concentrations of MDK and AGR2 were not significantly altered by tumor type or stage of disease. It is of note, however, that plasma concentrations of MDK and AGR2 display differential responsiveness in women with ovarian cancer when compared to CA125. In the case cohort, plasma concentrations of MDK but not AGR2 correlated significantly with CA125 concentrations. The lack of correlation between AGR2 and CA125 and AGR2 and midkine plasma concentrations in women with ovarian cancer may provide an opportunity to improve diagnostic efficiency by reducing the false negative rate and may be reflective of stage and/or tumor type- differential expression of AGR2 and CA125. This study, however, was not designed to definitively assess the relationship between analyte plasma concentration and disease stage and type and a larger cohort study would be required to resolve these relationships.

The diagnostic utility of MDK and AGR2 was further demonstrated by ROC curve analysis. It is acknowledged that good risk prediction models have an AUC > 0.7 [21]. The AUCs for MDK and AGR2 were 0.753 and 0.768, respectively. Individually, neither MDK nor AGR2 was superior to the classification efficiency of CA125 alone. In combination with CA125, however, MDK and AGR2 significantly increased AUC by more than 0.05 to greater than 0.98. Within the study cohort, the increased diagnostic efficiency of the multi-analyte algorithm reduced false positive and false negative rates by more than 50% when compared with CA125 alone. The sensitivity and specificity of the multi-analyte algorithm was 95.2 and 97.7%, respectively. It is of note that the performance of the three analyte algorithm developed in it this study, at least, is comparable to that of previously reported algorithms containing a greater number of biomarkers (*e.g. *[8]).

The involvement of both MDK and AGR2 in oncogenesis and tumor progression has been previously reported. MDK is a 13-kDa secreted heparin-binding growth factor [22,23], first identified in 1988 [24] and recent implicated in cell proliferation and survival, migration and angiogenesis [25-31]. Furthermore, MDK expression is induced in association with oncogenesis, inflammation and wound healing [32,33] and is over-expressed in various human cancers, including ovarian cancer [34-38] and may contribute to the development of chemotherapy drug resistance [39].

Anterior Gradient 2 protein is the protein product of a proto-oncogene (7p21.3) implicated in cell migration, differentiation and proliferation and is over-expressed in cancer of various origins. In human breast cancer cells, AGR2 expression correlates positively with estrogen receptor [40] and negatively with epidermal growth factor receptor expression [41]. These data are consistent with the hypothesis that AGR2 may play a role in the differentiation of hormonally responsive breast cancers [40,42]. More recently, a role for AGR2 in the aetiology of ovarian epithelial cancer has been proposed. AGR2 gene expression is significantly increased in ovarian carcinomas, particularly in mucinous tumors [43]. In non-neoplastic ovarian epithelial tissue, immunoreactive (ir) AGR2 was not detectable, however, virtually all ovarian tumors of variable histotypes stain positively for irAGR2. In addition, AGR2 has been reported to be released into the circulation of ovarian cancer patients [11]. Previous studies have reported that overexpression of AGR2 may promote the development of metastatic phenotype in benign breast cancer cell [42] and secreted AGR2 has been implicated in promoting proliferation of pancreatic cell lines in culture [44]. In addition, circulating tumor cells from patients with advanced metastatic disease display elevated AGR2 gene expression [45] suggesting that AGR2 may play a functional role in metastasis or may represent a useful biomarker of circulating tumor cells [46].

## Conclusion

The data obtained in this study confirm that the measurement of plasma concentrations of MDK and AGR2 individually display utility as biomarkers for ovarian cancer and that when included in a multi-analyte panel may significantly improve the diagnostic utility of CA125 in symptomatic women.

## Competing interests

TAE and DJA are all employees of Healthlinx Ltd, GR is non-executive chairman of Healthlinx Ltd.

## Authors' contributions

Only the authors were responsible for study design, analysis and interpretation of data, writing and submission of the manuscript for publication. All authors have read and approved the final version of this manuscript.

## References

[B1] Paley PJ Ovarian cancer screening: are we making any progress? Curr Opin Oncol 2001 13 399 402 10.1097/00001622-200109000-00015 11555720

[B2] Nossov V Amneus M Su F Lang J Janco JMT Reddy ST Farias-Eisner R The early detection of ovarian cancer: from traditional methods to proteomics. Can we really do better than serum CA-125? American Journal of Obstetrics and Gynecology 2008 199 215 223 10.1016/j.ajog.2008.04.009 18468571

[B3] Jacobs IJ Menon U Progress and challenges in screening for early detection of ovarian cancer Molecular & Cellular Proteomics 2004 3 355 366 10.1074/mcp.R400006-MCP200 14764655

[B4] Lokshin AE Yurkovetsky Z Bast R Lomakin A Maxwel GL Godwin AK Serum multimarker assay for early diagnosis of ovarian cancer Gynecologic Oncology 2008 108 S113 S114

[B5] Bertenshaw GP Yip P Seshaiah P Zhao J Chen TH Wiggins WS Mapes JP Mansfield BC Multianalyte profiling of serum antigens and autoimmune and infectious disease molecules to identify biomarkers dysregulated in epithelial ovarian cancer Cancer Epidemiology, Biomarkers & Prevention 2008 17 2872 2881 10.1158/1055-9965.EPI-08-0464 18843033

[B6] Nosov V Su F Amneus M Birrer M Robins T Kotlerman J Reddy S Farias-Eisner R Validation of serum biomarkers for detection of early-stage ovarian cancer American Journal of Obstetrics and Gynecology 2009 200 19285648 10.1016/j.ajog.2008.12.042

[B7] Zhang Z Bast RC Vergote I Hogdall C Ueland FR Van der Zee A Wang Z Yip C Chan DW Fung ET A large-scale multi-center independent validation study of a panel of seven biomarkers for the detection of ovarian cancer Journal of Clinical Oncology 2006 24 269S 269S

[B8] Edgell T Martin-Roussety G Barker G Autelitano DJ Allen D Grant P Rice GE Phase II biomarker trial of a multimarker diagnostic for ovarian cancer J Cancer Res Clin Oncology 2010 10.1007/s00432-009-0755-5 PMC2874491 20082099

[B9] Gorelik E Landsittel DP Marrangoni AM Modugno F Velikokhatnaya L Winans MT Bigbee WL Herberman RB Lokshin AE Multiplexed immunobead-based cytokine profiling for early detection of ovarian cancer Cancer Epidemiol Biomarkers Prev 2005 14 981 987 10.1158/1055-9965.EPI-04-0404 15824174

[B10] Visintin I Feng Z Longton G Ward DC Alvero AB Lai Y Tenthorey J Leiser A Flores-Saaib R Yu H Diagnostic markers for early detection of ovarian cancer Clin Cancer Res 2008 14 1065 1072 10.1158/1078-0432.CCR-07-1569 18258665

[B11] Edgell TA Barraclough DL Rajic A Dhulia J Lewis KJ Armes JE Barraclough R Rudland PS Rice GE Autelitano DJ Increased plasma concentrations of anterior gradent 2 protein are positively associated with ovarian cancer Clin Sci (Lond) 2010 in press 20136634 10.1042/CS20090537

[B12] Pepe MS Etzioni R Feng Z Potter JD Thompson ML Thornquist M Winget M Yasui Y Phases of biomarker development for early detection of cancer J Natl Cancer Inst 2001 93 1054 1056 10.1093/jnci/93.14.1054 11459866

[B13] Kruskal WH Wallis WA Use of ranks in one-criterion variance analysis Journal of the American Statistical Association 1952 47 583 621 10.2307/2280779

[B14] Dunn OJ Multiple comparisons using rank sums Technometrics 1964 6 241 10.2307/1266041

[B15] Witten IH Frank E Data Mining: Practical machine learning tools and techniques 2005 2 Morgan Kaufmann 2005: San Francisco

[B16] Hall M Frank E Holmes G Pfahringer B Reutemann P Witten IH The WEKA Data Mining Software: An Update; SIGKDD Explorations SIGKDD Explorations 2009 11

[B17] Waegeman W De Baets B Boullart L On the scalability of ordered multi-class ROC analysis Computational Statistics & Data Analysis 2008 52 3371 3388 10.1016/j.csda.2007.12.001

[B18] Hanley JA McNeil BJ A method of comparing the areas under receiver operating characteristic curves derived from the same cases Radiology 1983 148 839 843 6878708 10.1148/radiology.148.3.6878708

[B19] Friedman J Hastie T Tibshirani R Additive logistic regression: A statistical view of boosting Annals of Statistics 2000 28 337 374 10.1214/aos/1016218223

[B20] Salama RMH Muramatsu H Kobayashi H Nomura S Shigehiko M Muramatsu T Serum levels of midkine, a heparin-binding growth factor, increase in both malignant and benign gynecological tumors Reprod Immunol Biol 2006 21 64 70 10.3192/jsirib.21.64

[B21] May A Wang TJ Biomarkers for cardiovascular disease: challenges and future directions Trends Mol Med 2008 14 261 267 10.1016/j.molmed.2008.04.003 18487087

[B22] Vigny M Raulais D Puzenat N Duprez D Hartmann MP Jeanny JC Courtois Y Identification of a new heparin-binding protein localized within chick basement-membranes European Journal of Biochemistry 1989 186 733 740 10.1111/j.1432-1033.1989.tb15267.x 2558016

[B23] Tomomura M Kadomatsu K Nakamoto M Muramatsu H Kondoh H Imagawa K Muramatsu T A retinoic acid responsive gene, mk, produces a secreted protein with heparin binding-activity Biochemical and Biophysical Research Communications 1990 171 603 609 10.1016/0006-291X(90)91189-Y 2403350

[B24] Kadomatsu K Midkine, a heparin-binding growth factor: Its discovery and functions Seikagaku - Journal of Japanese Biochemical Society 1998 70 1315 1325 9889592

[B25] Muramatsu H Shirahama H Yonezawa S Maruta H Muramatsu T Midkine, a retinoic acid-inducible growth-differentiation factor - immunochemical evidence for the function and distribution Developmental Biology 1993 159 392 402 10.1006/dbio.1993.1250 8405666

[B26] Garver RI Radford DM Doniskeller H Wick MR Milner PG Midkine and pleiotrophin expression in normal and malignant breast-tissue Cancer 1994 74 1584 1590 10.1002/1097-0142(19940901)74:5<1584::AID-CNCR2820740514>3.0.CO;2-V 7520350

[B27] Choudhuri R Zhang HT Donnini S Ziche M Bicknell R An angiogenic role for the neurokines midkine and pleiotrophin in tumorigenesis Cancer Research 1997 57 1814 1819 9135027

[B28] Maeda N Ichihara-Tanaka K Kimura T Kadomatsu K Muramatsu T Noda M A receptor-like protein-tyrosine phosphatase PTP zeta/RPTP beta binds a heparin-binding growth factor midkine - Involvement of arginine 78 of midkine in the high affinity binding to PTP zeta Journal of Biological Chemistry 1999 274 12474 12479 10.1074/jbc.274.18.12474 10212223

[B29] Qi MS Ikematsu S Maeda N Ichihara-Tanaka K Sakuma S Noda M Muramatsu T Kadomatsu K Haptotactic migration induced by midkine - Involvement of protein-tyrosine phosphatase xi, mitogen-activated protein kinase, and phosphatidylinositol 3-kinase Journal of Biological Chemistry 2001 276 15868 15875 11340082 10.1074/jbc.m005911200

[B30] Zou P Muramatsu H Sone M Hayashi H Nakashima T Muramatsu T Mice doubly deficient in the midkine and pleiotrophin genes exhibit deficits in the expression of beta-tectorin gene and in auditory response Laboratory Investigation 2006 86 645 653 10.1038/labinvest.3700428 16619002

[B31] Owada K Sanjo N Kobayashi T Mizusawa H Muramatsu H Muramatsu T Michikawa M Midkine inhibits caspase dependent apoptosis via the activation of mitogen-activated protein kinase and phosphatidylinositol 3-kinase in cultured neurons Journal of Neurochemistry 1999 73 2084 2092 10537068

[B32] Yuki T Ishihara S Rumi MAK Ortega-Cava CF Kadowaki Y Kazumori H Ishimura N Amano Y Moriyama N Kinoshita Y Increased expression of midkine in the rat colon during healing of experimental colitis American Journal of Physiology-Gastrointestinal and Liver Physiology 2006 291 G735 G743 10.1152/ajpgi.00388.2005 16959957

[B33] Maruyama K Muramatsu H Ishiguro N Muramatsu T Midkine, a heparin-binding growth factor, is fundamentally involved in the pathogenesis of rheumatoid arthritis Arthritis and Rheumatism 2004 50 1420 1429 10.1002/art.20175 15146411

[B34] Abe Y Tsutsui T Mu J Kosugi A Yagita H Sobue K Niwa O Fujiwara H Hamaoka T A defect in cell-to-cell adhesion via integrin-fibronectin interactions in a highly metastatic tumor cell line Japanese Journal of Cancer Research 1997 88 64 71 9045898 10.1111/j.1349-7006.1997.tb00303.x PMC5921243

[B35] Nakanishi T Kadomatsu K Okamoto T Tomoda Y Muramatsu T Expression of midkine and pleiotropin in ovarian tumors Obstetrics and Gynecology 1997 90 285 290 10.1016/S0029-7844(97)00237-8 9241309

[B36] Maehara H Kaname T Yanagi K Hanzawa H Owan I Kinjou T Kadomatsu K Ikematsu S Iwamasa T Kanaya F Naritomi K Midkine as a novel target for antibody therapy in osteosarcoma Biochemical and Biophysical Research Communications 2007 358 757 762 10.1016/j.bbrc.2007.04.183 17506984

[B37] Tao P Xu DH Lin SB Ouyang GL Chang YD Chen Q Yuan YY Zhuo XM Luo QC Li J Abnormal expression, highly efficient detection and novel truncations of midkine in human tumors, cancers and cell lines Cancer Letters 2007 253 60 67 10.1016/j.canlet.2007.01.019 17379400

[B38] Ikematsu S Nakagawara A Nakamura Y Ohira M Shinjo M Kishida S Kadomatsu K Plasma midkine level is a prognostic factor for human neuroblastoma Cancer Science 2008 99 2070 2074 19016768 10.1111/j.1349-7006.2008.00957.x PMC11159802

[B39] Kang HC Kim IJ Park JH Shin Y Ku JL Jung MS Yoo BC Kim HK Park JG Identification of genes with differential expression in acquired drug-resistant gastric cancer cells using high-density oligonucleotide microarrays Clinical Cancer Research 2004 10 272 284 10.1158/1078-0432.CCR-1025-3 14734480

[B40] Thompson DA Weigel RJ hAG-2, the human homologue of the Xenopus laevis cement gland gene XAG-2, is coexpressed with estrogen receptor in breast cancer cell lines Biochemical and Biophysical Research Communications 1998 251 111 116 10.1006/bbrc.1998.9440 9790916

[B41] Fletcher GC Patel S Tyson K Adam PJ Schenker M Loader JA Daviet L Legrain P Parekh R Harris AL Terrett JA hAG-2 and hAG-3, human homologues of genes involved in differentiation, are associated with oestrogen receptor-positive breast tumors and interact with metastasis gene C4.4a and dystroglycan British Journal of Cancer 2003 88 579 585 10.1038/sj.bjc.6600740 12592373 PMC2377166

[B42] Liu D Rudland PS Sibson DR Platt-Higgins A Barraclough R Human homologue of cement gland protein, a novel metastasis inducer associated with breast carcinomas Cancer Research 2005 65 3796 3805 10.1158/0008-5472.CAN-04-3823 15867376

[B43] Marquez RT Baggerly KA Patterson AP Liu JS Broaddus R Frumovitz M Atkinson EN Smith DI Hartmann L Fishman D Patterns of gene expression in different histotypes of epithelial ovarian cancer correlate with those in normal fallopian tube, endometrium, and colon Clinical Cancer Research 2005 11 6116 6126 10.1158/1078-0432.CCR-04-2509 16144910

[B44] Ramachandran V Arumugam T Wang HM Logsdon CD Anterior gradient 2 is expressed and secreted during the development of pancreatic cancer and promotes cancer cell survival Cancer Research 2008 68 7811 7818 10.1158/0008-5472.CAN-08-1320 18829536 PMC4429896

[B45] Smirnov DA Zweitzig DR Foulk BW Miller MC Doyle GV Pienta KJ Meropol NJ Weiner LM Cohen SJ Moreno JG Global gene expression profiling of circulating tumor cells Cancer Research 2005 65 4993 4997 10.1158/0008-5472.CAN-04-4330 15958538

[B46] Valladares-Ayerbes M Diaz-Prado S Reboredo M Medina V Iglesias-Diaz P Lorenzo-Patino MJ Campelo RG Tch MH Tch IS Anton-Aparicio LM Bioinformatics approach to mRNA markers discovery for detection of circulating tumor cells in patients with gastrointestinal cancer Cancer Detection and Prevention 2008 32 236 250 10.1016/j.cdp.2008.08.002 18801625

